# Recombinant polymorphic membrane protein D in combination with a novel, second-generation lipid adjuvant protects against intra-vaginal *Chlamydia trachomatis* infection in mice

**DOI:** 10.1016/j.vaccine.2016.06.081

**Published:** 2016-07-29

**Authors:** Wayne Paes, Naj Brown, Andrzej M. Brzozowski, Rhea Coler, Steve Reed, Darrick Carter, Martin Bland, Paul M. Kaye, Charles J.N. Lacey

**Affiliations:** a Centre for Immunology and Infection, University of York, York YO10 5DD, United Kingdom; b York Structural Biology Laboratory, University of York, York YO10 5DD, United Kingdom; c Infectious Disease Research Institute, Seattle, WA 98102, United States; d Department of Health Sciences, University of York, York YO10 5DD, United Kingdom

**Keywords:** *Chlamydia trachomatis*, Vaccine, Pmps, TLR4 adjuvant

## Abstract

The development of a chlamydial vaccine that elicits protective mucosal immunity is of paramount importance in combatting the global spread of sexually transmitted *Chlamydia trachomatis* (*Ct*) infections. While the identification and prioritization of chlamydial antigens is a crucial prerequisite for efficacious vaccine design, it is likely that novel adjuvant development and selection will also play a pivotal role in the translational potential of preclinical *Ct* vaccines. Although the molecular nature of the immuno-modulatory component is of primary importance, adjuvant formulation and delivery systems may also govern vaccine efficacy and potency. Our study provides the first preclinical evaluation of recombinant *Ct* polymorphic membrane protein D (rPmpD) in combination with three different formulations of a novel second-generation lipid adjuvant (SLA). SLA was rationally designed *in silico* by modification of glucopyranosyl lipid adjuvant (GLA), a TLR4 agonistic precursor molecule currently in Phase II clinical development. We demonstrate robust protection against intra-vaginal *Ct* challenge in mice, evidenced by significantly enhanced resistance to infection and reduction in mean bacterial load. Strikingly, protection was found to correlate with the presence of robust anti-rPmpD serum and cervico-vaginal IgG titres, even in the absence of adjuvant-induced Th1-type cellular immune responses elicited by each SLA formulation, and we further show that anti-rPmpD antibodies recognize *Ct* EBs. These findings highlight the utility of SLA and rational molecular design of adjuvants in preclinical *Ct* vaccine development, but also suggest an important role for anti-rPmpD antibodies in protection against urogenital *Ct* infection.

## Highlights

• rPmpD in combination with SLA elicits significant protection against intra-vaginal *Ct* challenge.
• Antibodies induced by immunisation with rPmpD recognise *Ct* elementary bodies.
• SLA is a novel adjuvant class that may be widely used in future preclinical *Ct* vaccine development.

## 1 Introduction

*Chlamydia trachomatis* (*Ct*) is the most common sexually transmitted bacterial pathogen worldwide, responsible for ∼131 million new cases of disease each year [1]. Urogenital infections are asymptomatic in 30–50% of men and 70–90% of women, with major complications such as pelvic inflammatory disease (PID) and infertility occurring predominantly in women [2]. Aggressive ‘seek and treat’ public health measures have not stemmed the rise of infection rates, leading to proposal of the ‘arrested immunity’ hypothesis [3]. This suggests vaccination as a key step in controlling and potentially eliminating *Ct* infections [4]. Due to the paucity of robust clinical data, protective immunological parameters have largely been derived from preclinical murine models.

*Ct* or the mouse pneumonitis biovar *Chlamydia muridarum* (*Cm*) have been utilised to study chlamydial infections and vaccine efficacy in mice, although neither model mimics all aspects of human disease and pathogenesis [5]. Several studies investigating protective immunity and vaccine efficacy have focused on intra-vaginal *Cm* challenge which typically results in a more prolific ascending infection with lower inoculating doses than *Ct*, that can rapidly progress to hydrosalpinx and infertility [6]. Thus, the *Cm* model may be widely applicable for the study of therapeutic vaccines against post-infection sequelae, although intra-vaginal infection with certain *Ct* serovars can also result in ascending infection [7]. Furthermore, it has been suggested that *Ct* infection in mice may more closely mimic the self-limiting infections in women that rarely progress so quickly to upper-genital tract pathology [8].

Although a high degree of genomic synteny is apparent, differences within the plasticity zones of *Cm* and *Ct* genomes may influence infection outcomes in the murine model [9], [10]. *Cm* possesses three functional paralogous copies of the cytotoxin gene that is truncated and likely non-functional in the majority of urogenital *Ct* serovars [11]. The cytotoxin gene is thought to influence chlamydial sensitivity to IFNγ through targeting of GTPases, and may differentially mediate innate immune evasion in the host [12], [13]. Such observations have cautionary implications for the investigation of chlamydial infections in mice, as it has recently been suggested that innate immunity is sufficient to resolve *Ct* but not *Cm* infection [14]. However, the study also indicates a dual role for adaptive immunity in reducing *Ct* bacterial burden and time to clearance, and vaccine-induced protective immunity has previously been investigated in mice using *Ct* as the agent of infection [15], [16], [17].

Subunit vaccines have displaced the use of whole-cell organisms following the incidence of inflammatory reactions in non-human primates post challenge, although this was found not to be the case in humans [18], [19], [20]. Hence, identification and prioritization of novel chlamydial antigens that elicit protective cell-mediated and/or humoral immunity is of great importance. Recent studies using *Cm* have highlighted a promising role for chlamydial polymorphic membrane proteins (Pmps) as vaccine candidates [21], [22]. However, Pmps E and F are highly polymorphic within genital *Ct* strains, and PmpG possesses regulatory sequences indicative of phase variation, suggesting a role for Pmps in immune evasion [23]. In contrast, *Ct* PmpD possesses the highest inter-strain sequence conservation (>99.15%) among the nine-member family, implying a conserved role in biphasic development or virulence [24]. Furthermore, PmpD has been implicated in mediating early host-cell interactions [25], [26].

Here, we provide the first preclinical investigation of a candidate chlamydial vaccine, evaluating three different formulations of a rationally designed TLR4 agonistic second-generation lipid adjuvant (SLA) in combination with *Ct* rPmpD. SLA was designed *in silico* (Carter et al., submitted) through rational modification of the terminal acyl chains of glucopyranosyl lipid adjuvant (GLA) [27], a precursor molecule that has demonstrated tolerability and immunogenicity in Phase 1 trials [28].

We demonstrate robust protection against urogenital *Ct* infection in C57BL/6 mice, characterized by significantly enhanced resistance to infection and bacterial clearance. While all SLA formulations elicited significantly enhanced magnitudes of rPmpD-specific Th1-biased immune responses that correlated with resistance to infection and reduced bacterial burden, protection was also observed following immunization with rPmpD alone in the absence of SLA-induced Th1-bias, which coincided with robust anti-rPmpD serum and cervico-vaginal IgG titres. Hence, we propose that anti-rPmpD antibodies may play a significant role in vaccine-mediated protection against intra-vaginal *Ct* challenge, and demonstrate that SLA represents a novel adjuvant class that may be more widely utilised in future chlamydial vaccine development.

## 2 Materials and methods

### 2.1 *Ct* cell culture

*Ct* serovar E/Bour (American Type Culture Collection [ATCC]) and *Ct* serovar D/UW3/Cx (Prof. Shattock, Imperial College) were propagated in McCoy cells (ATCC) and purified as described previously [29].

### 2.2 Preparation of recombinant PmpD and UV-inactivated *Ct* EB vaccines

A DNA construct encoding the ∼65 kDa (aa68-aa698) passenger domain fragment previously identified by mass spectrometry following in vitro infection [30] was inserted into the pET 28(b) vector (Novagen) with a C-terminal hexa-histidine tag. Following over-expression in *E. coli* BL21(DE3) strain (Novagen), purification and refolding was implemented by stepwise dialysis of 6 M guanidinium hydrochloride from inclusion bodies, ending up in 20 mM Tris, 50 mM NaCl (pH 8). This yielded soluble protein that was purified by metal affinity and size exclusion chromatography. Following 0.1% Triton X-114 extraction of *E. coli* LPS [31], the Limulus Amebocyte Lysate test was utilised to determine the amount of endotoxin in the rPmpD preparation, which was found to be below 1EU/mg. *Ct* E/Bour elementary bodies were inactivated by UV irradiation (UVEB) at a distance of 15 cm from a UV-lamp for 60 min.

### 2.3 Second-generation lipid adjuvant formulations

Second-generation lipid adjuvant (SLA) was synthesised by Corden Pharma (Liestal, Switzerland). Aqueous formulations (SLA-AF) were manufactured by mixing SLA with dipalmitoyl phosphatidylcholine (DPPC) at a DPPC:SLA molar ratio of 2:1 in chloroform, and evaporated. Ultra-pure water was added, and the mixture sonicated in a 60 °C water bath until translucent. Oil-in-water emulsions (SLA-SE) were manufactured by high-speed mixing poloxamer 188, glycerol, and ammonium phosphate buffer with an oil phase (squalene, dimyristoyl phosphatidylcholine) and SLA. The liposomal formulation (SLA-LSQ) was mixed with aqueous QS21 following addition of dioleoyl phosphatidylcholine:cholesterol (4:1 w/w ratio) with SLA in chloroform and hydration in 25 mM ammonium phosphate buffer (pH ∼5.7) with sonication to achieve a monodisperse particle size ∼80 nm.

### 2.4 Mouse immunizations

All animal care and experimental procedures were performed under UK Home Office License (Ref # PPL 70/7805) and with approval from the Animal Procedures and Ethics Committee of the University of York. 6–8 week old female C57BL/6 mice were bred and maintained under specific pathogen-free conditions at the University of York, allocated to groups and immunized subcutaneously (s.c.) at the base of the tail on days 0, 21 and 42 with 100 μl of sterile PBS containing 5 μg/dose of adjuvant (SLA-AF, SLA-SE or SLA-LSQ) with 5 μg/dose rPmpD or 2 × 10^7^ inclusion forming units (IFU) of UVEBs. Additional groups of mice received rPmpD, SLA-AF, SLA-SE, SLA-LSQ or PBS alone as controls.

### 2.5 Vaginal *Ct* challenge and determination of chlamydial load

All animals received a subcutaneous injection of 2.5 mg of medroxyprogesterone acetate (Depo-Provera, Pfizer) one week prior to challenge. Three weeks after the final vaccination, mice were challenged intra-vaginally with 10^7^ IFU of *Ct* serovar D/UW3/Cx EBs as previously described [7], [17]. Cervico-vaginal swabs were obtained on days 1, 3, 7, 14 and 22 post-challenge, and the total number of inclusions per well were enumerated in a blinded fashion using fluorescence microscopy.

### 2.6 Cervico-vaginal washes

Cervico-vaginal washes were obtained on days 34 and 55 post initial immunization. 100 μl of sterile-filtered PBS were pipetted up-and-down the vaginal cavity ten times using a Microman Precision Microliter Pipette with rounded-tip capillary pistons (Gilson). To prevent protein degradation, 25× Protease Inhibitor Cocktail (Sigma–Aldrich) was added. Cervico-vaginal washes were stored at −80 °C until use.

### 2.7 Measurement of serum and cervico-vaginal antigen-specific antibody

Serum was obtained on days 0, 35 and 56 following initial immunization and 6 weeks post challenge. 96-well Assay plates (Costar) were coated with 50 μl rPmpD (1 μg/ml) at 4 °C overnight in coating buffer (0.1 M Na_2_CO_3_, 0.1 M NaHCO_3_, pH 9.6). Plates were washed four times with 300 μl/well wash buffer (1× PBS, 0.05% Tween-20), and all subsequent washing steps carried out in identical fashion using an automated SkanWasher 400 (Molecular Devices, USA). Blocking buffer (1× PBS, 0.05% Tween-20, 0.1% BSA) was added (200 μl/well) and plates incubated for 2hr at room temperature prior to addition of serial dilutions of serum (100 μl/well) or cervico-vaginal lavage. After incubation for 2 h at 37 °C, plates were washed and then incubated with HRP-conjugated goat anti-mouse IgG, IgG1 or IgG2c antibodies (AbD Serotec) at 37 °C for 30 minutes. After the final wash step, 50 μl/well of 3′,5,5′-tetramethylbenzidine (TMB) substrate solution (Sigma-Aldrich) was added. Colour development was stopped after 5 min with 1 M H_2_SO_4_ (KPL, USA) and plates were read immediately at 450 nm (OD_450_) with a VersaMax microplate reader in conjunction with the SoftMax Pro v.5.3 software (Molecular Devices, USA). Reciprocal serum dilutions corresponding to 50% maximal binding were used to obtain titres.

### 2.8 Antigen-specific cellular responses

Animals were euthanized and spleens collected and homogenized into single cell suspensions. Enzyme-linked immunospot (ELISpot) assays were performed immediately following splenic harvest. Briefly, 96-well MultiScreen filtration plates (Millipore) were coated overnight at 4 °C with IFNγ, IL-5 or IL-10 monoclonal capture antibodies (Mabtech). Splenocytes (2.5 × 10^5^ cells/well) were added to the plate in the presence of 1 μg/ml rPmpD. After incubation at 37 °C with 5% CO_2_ for 20 h, plates were washed and incubated with respective biotinylated detection antibodies according to manufacturer’s protocol (Mabtech). This was followed by incubation with streptavidin-alkaline phosphatase (1 h, RT) and addition of BCIP/NBT-plus substrate until distinct spots emerged. Spot forming units (SFU) were enumerated by eye.

### 2.9 Statistical analysis

Differences between two independent means were determined with a two-tailed t-test using GraphPad Prism 6. For multiple comparisons, groups were compared using a one-way analysis of variance (ANOVA) following transformation of skewed distributions in STATA/IC 10 (StataCorp, USA). Sample size calculations were performed in G∗Power version 3.1 using both *a priori* analysis based on previous studies [17], [22] and *post hoc* pilot experiments performed in our laboratory. A total of 5 animals were sufficient to detect expected effect sizes with a power of 0·8 (alpha = 0·05) for all experimental readouts.

## 3 Results

### 3.1 SLA in combination with rPmpD induces a robust Th1-type immune response and significantly enhances systemic and cervico-vaginal rPmpD-specific IgG titres

**Fig. 1:**
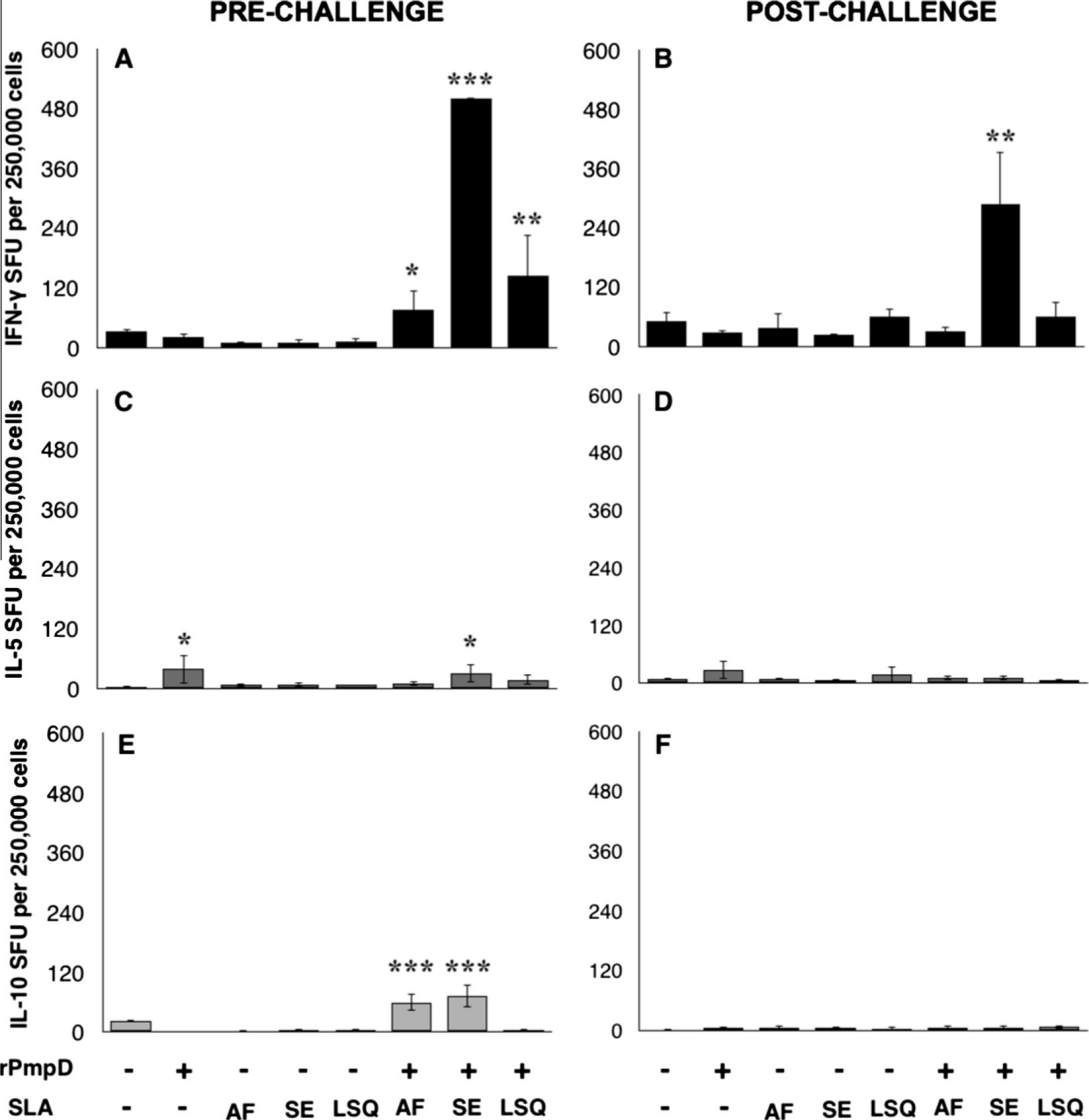
rPmpD-specfic cytokine responses following immunization with different vaccine formulations in C57BL/6 mice. Splenocytes were harvested and stimulated with rPmpD two weeks after the final immunization (A, C, E) or six weeks post-challenge (B, D, F), and assessed for IFN_Y_, IL-5 or IL-10 secretion. SLA formulation-specific differences in cytokine profiles are observed. The results are expressed as the mean ± the standard deviation for groups of 5 mice measured in triplicate from two experiments (^*^P⩽0.05, ^**^P⩽0.01, ^***^P⩽0.001 relative to PBS-immunized mice).

To evaluate the immunogenicity of rPmpD adjuvanted with different formulations of SLA, C57BL/6 mice were immunized, and splenic rPmpD-specific cellular immune responses elicited by each vaccine formulation were determined using ELISpot both pre- and post-*Ct* challenge (Fig. 1A–F). In the absence of adjuvant, immunization with rPmpD elicited a minimal recall IFNγ response, with no detectable significant difference from control groups (Fig. 1A). However, mice immunized with rPmpD in combination with all SLA formulations showed significantly elevated antigen-specific IFNγ responses relative to all control groups (*p* ⩽ 0.05). Formulation-specific differences in the magnitude of the T cell responses were observed. SLA-SE elicited the most robust IFNγ response following stimulation with rPmpD (>500 spots/250,000 spleen cells), with no significant difference between IFNγ levels in SLA-AF-immunized (76 ± 38 cells/250,000) and SLA-LSQ-immunized mice (145 ± 81/250,000) detected (*p* = 0.1739). Although the rPmpD-specific IL-10 response was significantly augmented with the SLA-SE and SLA-AF formulations (Fig. 1E), the significantly enhanced IFNγ:IL-10 ratio elicited by the SLA-SE-rPmpD vaccine clearly indicates that SLA-SE biases towards robust Th1-type immune responses in vivo. Interestingly, only the SLA-SE-induced rPmpD-specific IFNγ response was significantly boosted following *Ct* infection (Fig. 1B).

**Fig. 2:**
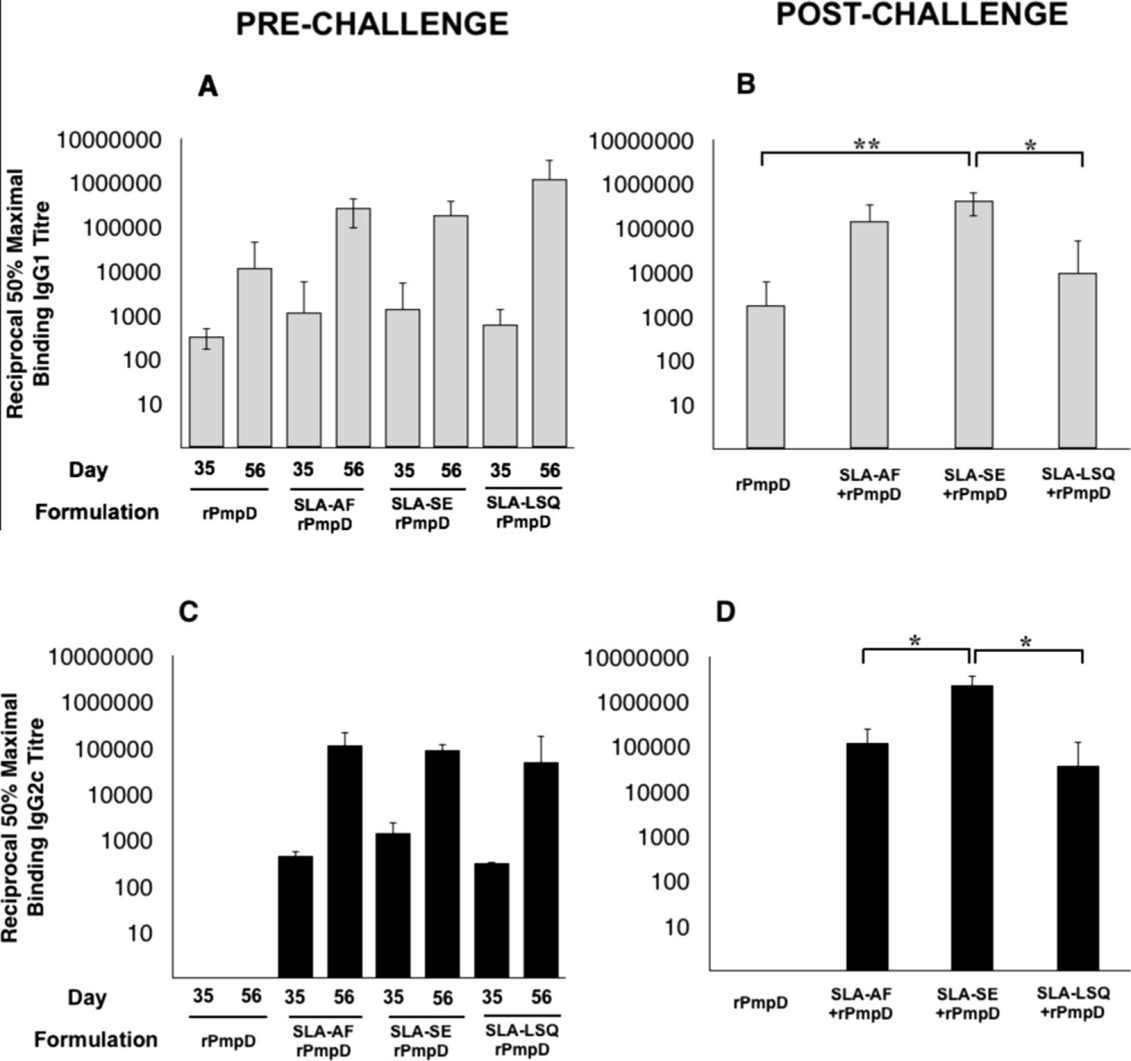
rPmpD-specific IgG1 and IgG2c responses following immunization with different rPmpD vaccine formulations in C57BL/6 mice. Anti-rPmpD serum IgG1 and IgG2c titres were measured two weeks following penultimate and final immunisations (A, C) or six weeks post-challenge (B, D). Results are expressed as the geometric mean ± the standard deviation for groups of 5 mice measured in triplicate, and are representative of two separate experiments. (^*^P⩽0.05, ^**^P⩽0.01).

To assess vaccine-induced humoral immunity, rPmpD-specific serum IgG responses were measured two weeks following the penultimate and final boosts (Fig. 2A and C). The reciprocal titres at 50% maximal binding of anti-rPmpD IgG1 and IgG2c were measured. No rPmpD-specific IgG was detected in mice immunized with PBS or SLA alone, while rPmpD administered in the absence of SLA elicited robust systemic IgG1 humoral immune responses, but showed no detectable IgG of the 2c subclass pre- or post-challenge (Fig. 2C and D). As class switching to IgG2c is mediated by IFNγ, these data are consistent with the cytokine results shown in Fig. 1. All rPmpD-SLA combinations induced robust IgG1 and IgG2c rPmpD-specific titres, with demonstrable increases in titres following successive immunizations that also remained elevated 6 weeks post infection with *Ct* (Fig. 2B and D).

**Fig. 3:**
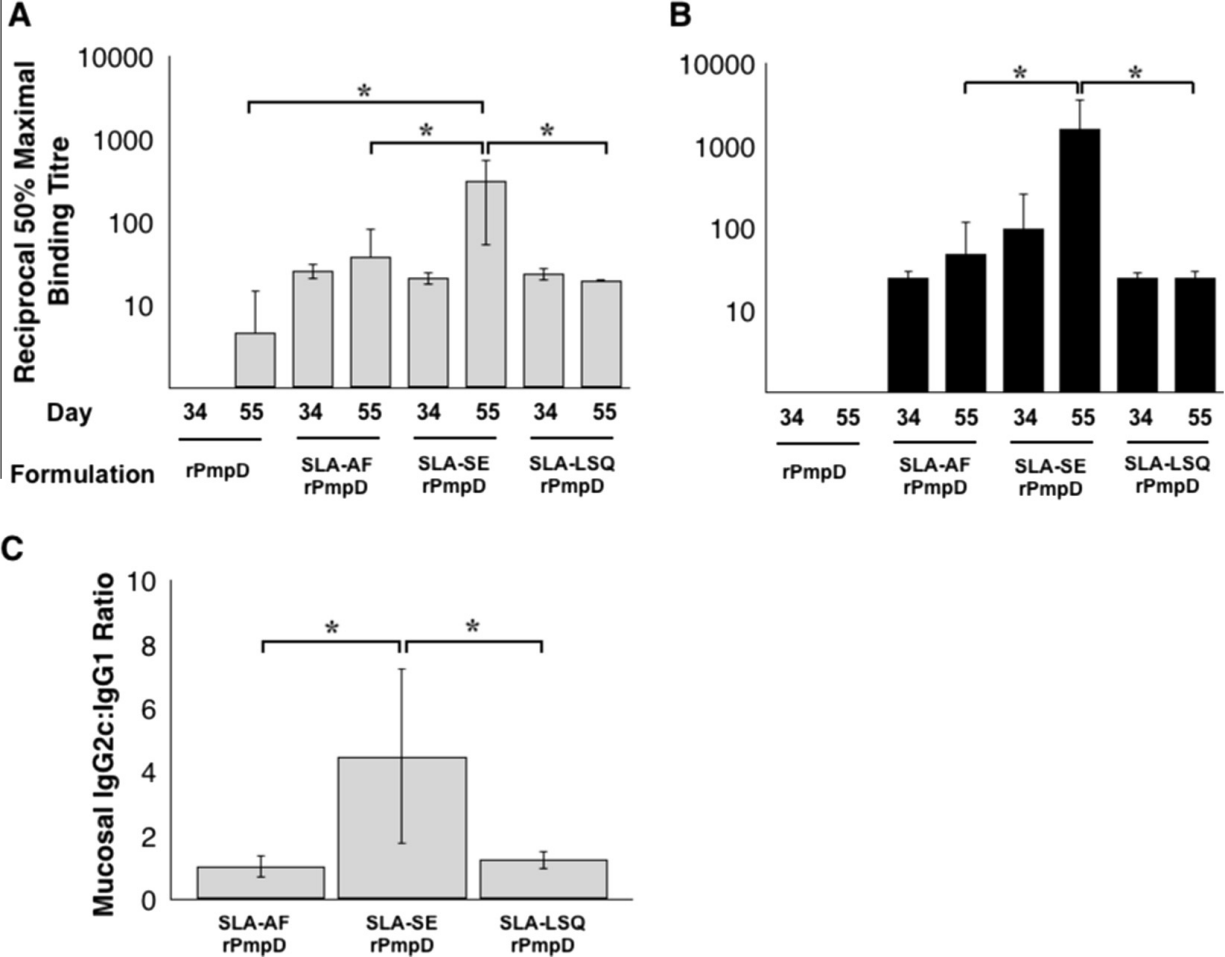
Immunization with rPmpD elicits robust cervico-vaginal antigen-specfic IgG titres. C57BL/6 mice were immunized with all rPmpD formulations and mucosal anti-rPmpD IgG1 (A) and IgC2c (B) titres were measured at the indicated time points prior to *Ct* challenge. (C) Formulation-specific IgG2c:IgG1 titre ratios two weeks post final immunization prior to *Ct* challenge. Results are expressed as the geometric mean ± the standard deviation for groups of 5 mice measured in triplicate for the same animals as in Fig. 2, and are representative of two separate experiments (^*^P⩽0.05).

To assess mucosal humoral responses, cervico-vaginal washes were obtained two weeks following the penultimate and final immunizations. Consistent with serum IgG subclasses, no detectable levels of IgG2c were observed in mice immunized with rPmpD alone. While no significant difference between SLA-AF and SLA-LSQ-induced cervico-vaginal titres were observed, the SLA-SE formulation elicited significantly greater anti-rPmpD IgG titres and IgG2c:IgG1 ratios within the vaginal vault following the final immunization (Fig. 3), consistent with robust induction of Th1-type immunity (Fig. 1A).

### 3.2 Reactivity of anti-rPmpD and anti-UVEB serum in Western Blot and ELISA

**Fig. 4:**
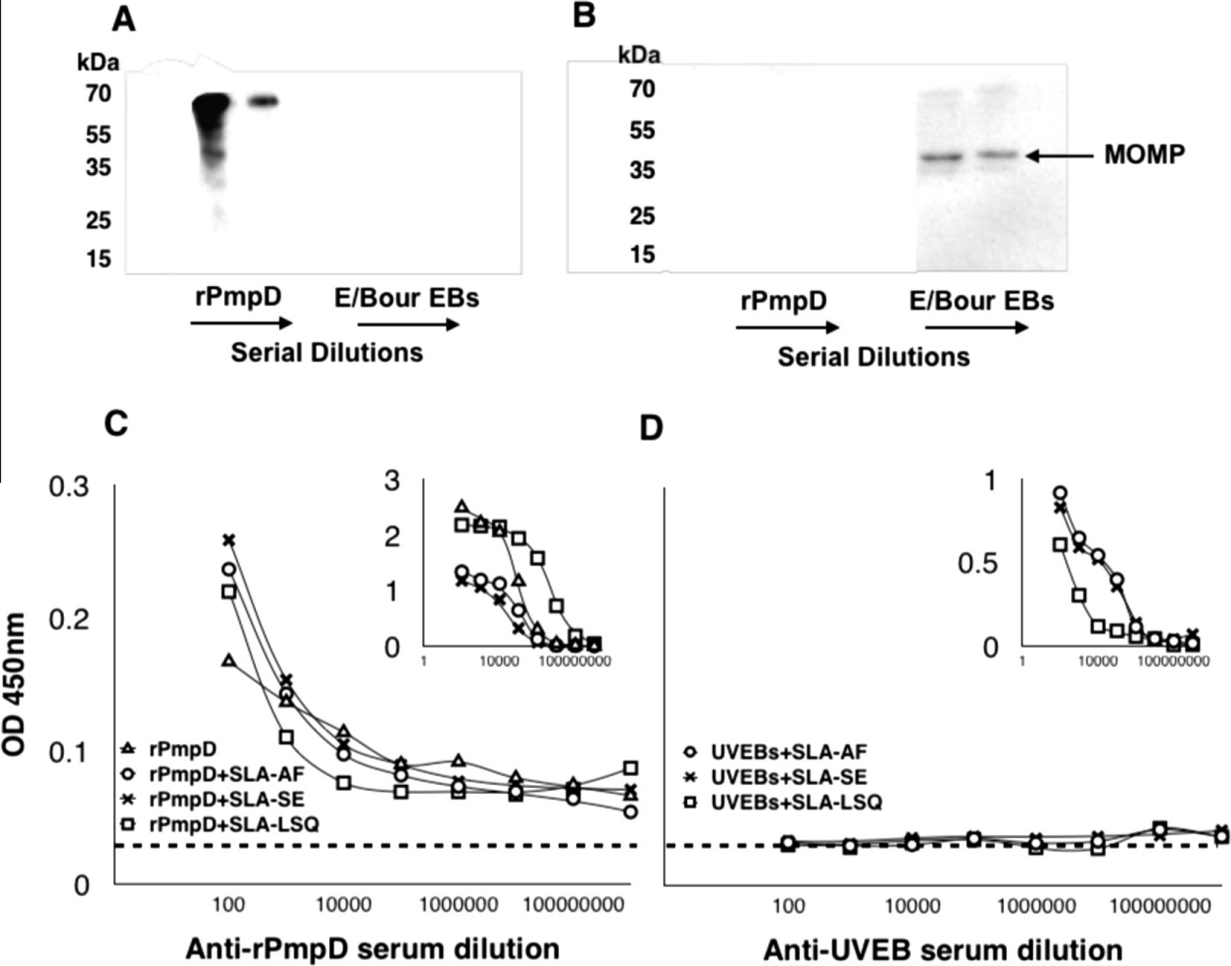
Reactivity of anti-rPmpD and anti-UVEB serum. Serum from mice immunized with rPmpD (A) or UVEBs (B) was tested for reactivity against each antigen in western blots. (C) Anti-rPmpD serum reacts with UVEBs in an indirect ELISA (inset shows reactivity against rPmpD). (D) Anti-UVEB serum failed to react with rPmpD (inset shows reactivity against UVEBs). Dotted lines represent the OD_450_ cut-off value equivalent to the mean plus two standard deviations of control pre-immune serum.

To determine reactivity against both native and non conformational-dependent epitopes, ELISA and western blotting were implemented using heat-inactivated serum from rPmpD- and UVEB-immunized mice two weeks following the final boost, prior to challenge (Fig. 4). Anti-rPmpD serum from all vaccine groups reacted strongly with rPmpD in both assays, and cross-reacted with UVEBs in ELISAs, showing recognition of epitopes on *Ct* EBs. Anti-UVEB serum did not cross-react with rPmpD in ELISA or western blot, but reacted with UVEBs in both assays, showing immunodominant recognition of *Ct* major outer membrane protein (MOMP) (Fig. 4B).

### 3.3 Vaccination with rPmpD in the presence or absence of SLA significantly enhances resistance to *Ct* infection and reduces mean bacterial load

**Fig. 5:**
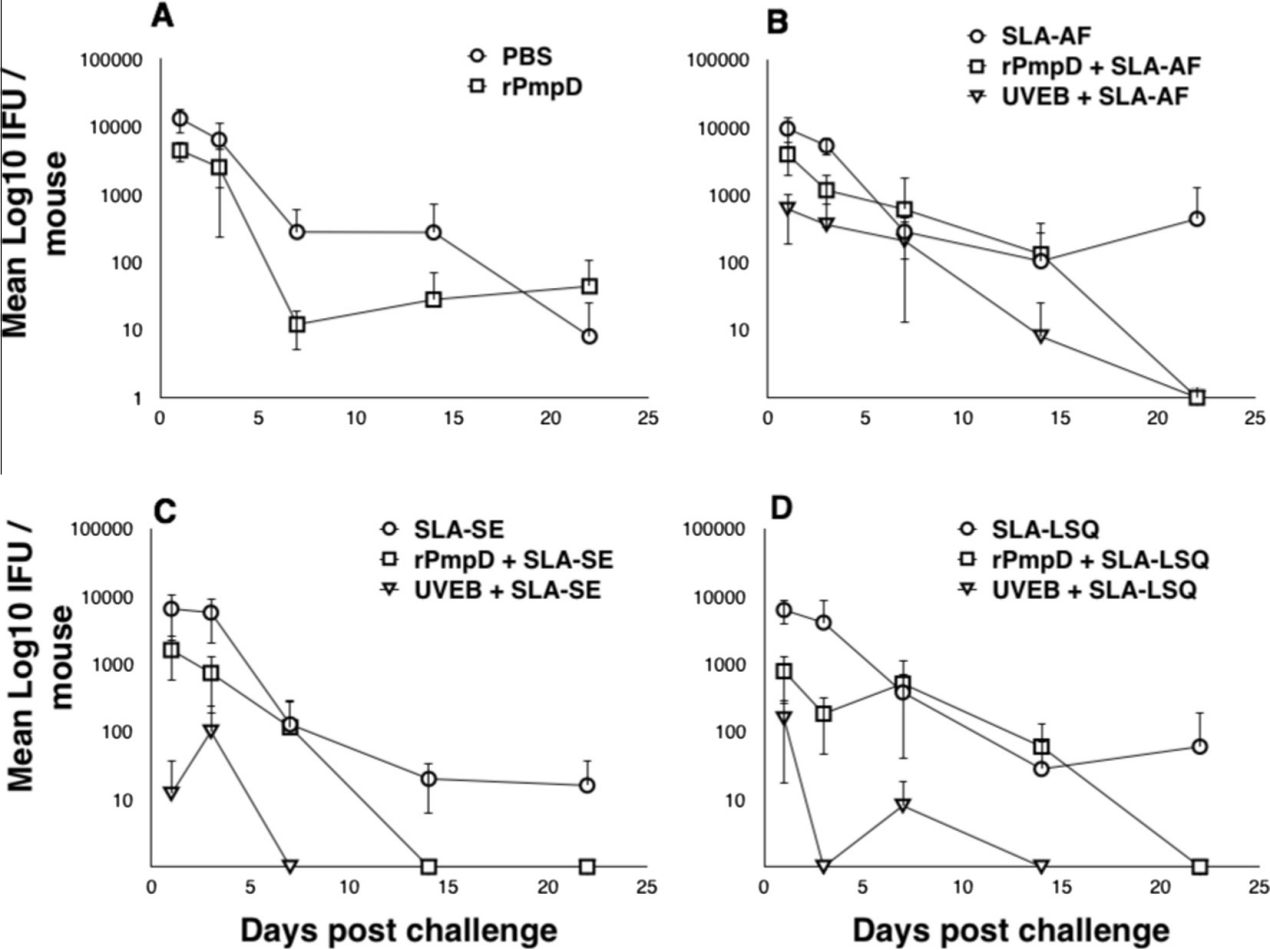
Infection curves showing bacterial shedding following challenge of vaccinated mice. C57BL/6 mice were infected intra-vaginally with *Ct* serovar D/UW3/Cx three weeks post final immunization with either rPmpD or *Ct* serovar E/Bour UVEBs (positive control). Individuals were swabbed on days 1, 3, 7, 14 and 22 post infection, and recoverable cervico-vaginal IFU assessed on Hak cell monolayers. Culture-negative mice were assigned a cut-off value of <10 IFU. Formualtion- and antigen-specific differences in time to complete resolution of infection are observed. Results are expressed as the mean ± the standard deviation for groups of 5 mice.

**Fig. 6:**
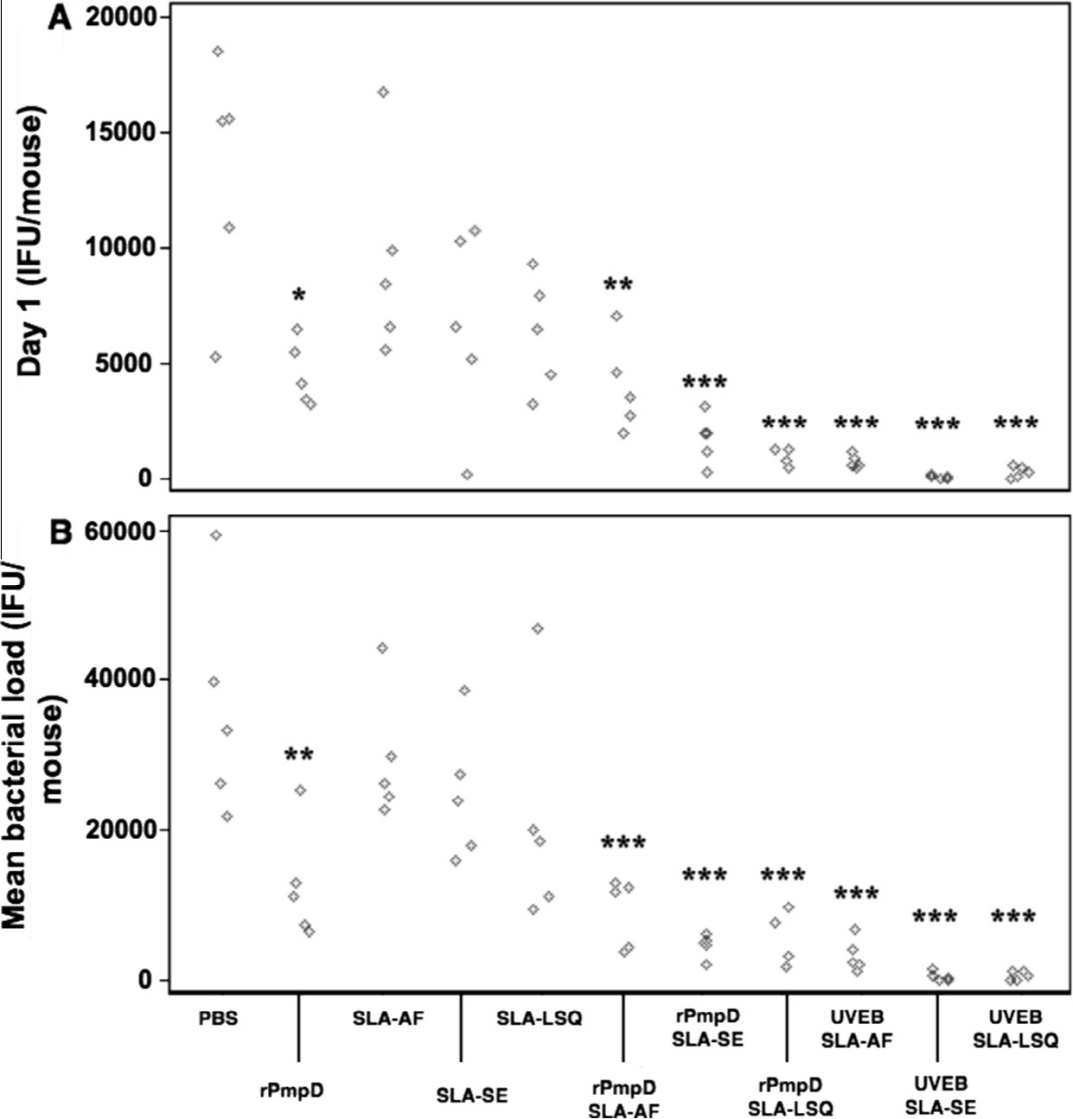
Quantification of vaccine elicited protection against *Ct* challenge. (A) Resistance to infection was assessed by swabbing individuals on day 1 and determining recoverable IFU on Hak cell monolayers. (B) Total bacterial load over the 22 day time period was quantified by integrating the area under the shedding curves (depicted in Fig. 5) for individual mice. Data were analysed using a one-way analysis of variance (ANOVA) for groups of five mice. Significance values displayed are relative to PBS-immunized mice (^*^P⩽0.05, ^**^P⩽0.01, ^***^P⩽0.001).

Cervico-vaginal swabs were obtained from all vaccine groups on days 1, 3, 7, 14 and 22 following intra-vaginal challenge with *Ct* serovar D (Fig. 5). For quantification of resistance to infection, recoverable IFU at day 1 were compared between each group using a one-way ANOVA (Fig. 6A). Mean bacterial load over time was quantified using an integrative area under the curve (AUC) method (Fig. 6B). All antigen-adjuvant combinations elicited enhanced resistance to infection and significant reduction in bacterial burden relative to sham-immunized mice (Fig. 6).

No significant differences in resistance to infection or bacterial burden between PBS-immunized mice and adjuvant only groups (SLA-AF, SLA-SE, SLA-LSQ) were observed, suggesting non-specific innate immune activation by SLA formulations in the absence of antigen does not play a role in protection. Furthermore, the time to complete clearance (where all individuals within a group were culture-negative) for each vaccine antigen was found to be formulation-dependent, with SLA-SE resulting in the shortest time to clearance relative to SLA-AF or SLA-LSQ formulations independent of antigen (Fig. 5C). Strikingly, rPmpD administered in the absence of SLA resulted in significantly enhanced resistance to infection and reduced bacterial load relative to sham-immunized mice (Fig. 6), although all individuals within this group failed to completely resolve infection (Fig. 5A). Although no significant difference in resistance to infection or bacterial load was observed between rPmpD-SLA vaccine formulations (Fig. 6), the time to clearance (Fig. 5) and magnitude of Th1 bias (Fig. 1A) were formulation-dependent. SLA-SE elicited the most robust rPmpD-specific IFNγ recall responses pre- and post-challenge, and resulted in the shortest time to clearance when coupled with either rPmpD or UVEBs (Fig. 5).

## 4 Discussion

Our study demonstrates the formulation-specific efficacy of SLA, a novel TLR4 agonist, at inducing Th1-biased immune responses and protection against *Ct* infection in combination with rPmpD in vivo. The enhanced magnitude of both innate and adaptive cellular responses observed with squalene emulsions (SE) has previously been associated with inflammasome activation in vivo. Early IFNγ and IL-18 production by neutrophils and memory CD8^+^ T cells was ablated in caspase-1/11^–/–^ and IL-18 receptor 1^–/–^ mice following administration of GLA-SE, thus proposing a mechanistic basis for the observed potency of SLA-SE in our study [32].

Importantly, we also highlight the protective efficacy of rPmpD against urogenital *Ct* infection in the absence of adjuvant. A similar phenomenon has recently been observed where un-adjuvanted recombinant *Ct* MOMP elicited comparable antibody responses to adjuvanted MOMP, which correlated with protection [33]. Robust pathogen-specific Th1-type immunity is widely regarded as a robust parameter for screening preclinical chlamydial vaccines [34], and Stary et al. have recently detailed cell-mediated effector mechanisms underlying vaccine-induced mucosal immunity against *Ct* infections [35]. Unexpectedly, despite the generation of robust *Ct*-specific humoral responses, the authors reported no definitive role for antibody-mediated protection, and detect no significant difference in recoverable IFU between immunized and naïve mice at day 1 post challenge. In contrast, we demonstrate significantly reduced recoverable IFU on day 1 between rPmpD- and sham-immunized mice (Fig. 6A).

Although possible vaccine-induced innate effector mechanisms cannot be ruled out, protection in our study correlates with the presence of robust pathogen-specific serum and mucosal antibody titres irrespective of SLA-induced Th1-bias. However, mice immunized with rPmpD alone fail to completely resolve infection at day 22 (Fig. 5A), suggesting that adaptive rPmpD-specific cellular immune responses may be important at enhancing bacterial clearance. Very few studies have highlighted a role for *Ct*-specific antibodies in vaccine-mediated protection, although antibody-mediated protection has previously been described in the *Cm* model of infection [36]. Indeed, the only convincing evidence describing a protective role for anti-*Ct* antibodies in vivo focuses on the antigenically variable MOMP antigen, where passive transfer of serum from immunized mice conferred early protection against infection, suggesting systemic neutralizing antibody is sufficient to mediate lower genital tract protection [37]. Our study demonstrates the efficacy of subcutaneous vaccination at eliciting robust antigen-specific mucosal IgG responses, and we further show that immune serum from rPmpD-immunized mice cross-reacts with UVEBs in ELISA (Fig. 4C), confirming antibody recognition of the agent of infection.

Further work is now required to detail the underpinnings of other likely immuno-modulatory properties of rPmpD. For instance, the *Leishmania major* hydrophilic acylated surface protein B (HASPB) elicited long-term protection and antigen-specific CD8^+^ T cells in the absence of exogenous adjuvant or delivery vehicle, where natural antibody binding and complement act as endogenous adjuvants that bridge innate and adaptive immunity [38]. Similarly, the leishmanial vaccine candidate antigen LeIF was shown to possess inherent IL-12 stimulating capacity [39], [40].

A prophylactic chlamydial vaccine that reduces infectious burden or time to clearance is likely to have significant global healthcare benefits. Based on our results and the efficacy of precursor molecule GLA in clinical trials, we propose that SLA may be incorporated as a novel adjuvant in future preclinical chlamydial vaccines with significant translational potential. Furthermore, the high degree of pan-strain conservation of *Ct* PmpD and immunodominance in patient cohorts highlights the potential for eliciting broad protection against a diverse array of circulating strains [24], [41], [42].

## Financial support

This research was supported by a Wellcome Trust PhD studentship to Wayne Paes (Grant 097325/Z/11/Z) and the Bill and Melinda Gates Foundation (Grants #42387 and OPP1055855) to SGR and DC. This research was also funded in whole or in part with Federal funds from the National Institute of Allergy and Infectious Diseases National Institutes of Health, Department of Health and Human Services, under Contract No. HHSN272200800045C and Grant No, U01AI078054 to RC. The content of this publication is solely the responsibility of the authors and does not necessarily reflect the views or policies of the Department of Health and Human Services, nor does mention of trade names, commercial products, or organizations imply endorsement by the U.S. Government.

## Potential conflicts of interest

SGR is a founder of, and holds an equity interest in, Immune Design, a licensee of certain rights associated with GLA and SLA. SGR and DC are inventors on issued patents for GLA based formulations and on SLA.
